# Finding low-complexity DNA sequences with longdust

**Published:** 2025-09-09

**Authors:** Heng Li, Brian Li

**Affiliations:** 1 Department of Biomedical Informatics, Harvard Medical School, 10 Shattuck St, Boston, MA 02215, USA; 2 Department of Data Science, Dana-Farber Cancer Institute, 450 Brookline Ave, Boston, MA 02215, USA; 3 Broad Insitute of MIT and Harvard, 415 Main St, Cambridge, MA 02142, USA; 4 Commonwealth School, Boston, MA 02116, USA

## Abstract

**Motivation:**

Low-complexity (LC) DNA sequences are compositionally repetitive sequences that are often associated with increased variant density and variant calling artifacts. While algorithms for identifying LC sequences exist, they either lack rigorous mathematical foundation or are inefficient with long context windows.

**Results:**

Longdust is a new algorithm that efficiently identifies long LC sequences including centromeric satellite and tandem repeats with moderately long motifs. It defines string complexity by statistically modeling the k-mer count distribution with the parameters: the k-mer length, the context window size and a threshold on complexity. Longdust exhibits high performance on real data and high consistency with existing methods.

**Availability and implementation:**

https://github.com/lh3/longdust

## 1. Introduction

In computer science, a string is of low complexity (LC) if it is repetitive in composition. LC strings tend to be tandemly repetitive and most of them can be identified with tandem repeat finding algorithms such as TRF (Benson, 1999), TANTAN (Frith, 2011), ULTRA (Olson and Wheeler, 2024) and pytrf (Du et al., 2025). These algorithms do not rigorously define string complexity. They rely on heuristics to search for impure tandem repeats and cannot identify LC strings without clear tandem structures. SDUST (Morgulis et al., 2006) is the only widely used algorithm that explicitly defines string complexity and finds the exact solutions. However, with $O\left(w^{3}L\right)$ time complexity, where w is the window size and L is the genome length, SDUST is inefficient given a large w and is thus impractical for finding satellite or tandem repeats with long motifs. Furthermore, the complexity scoring function used by SDUST is not backed by rigorous modeling. The theoretical properties of LC strings defined this way are unclear.

Inspired by SDUST, we sought an alternative way to define the k-mer complexity of a string and to identify LC regions in a genome. Our complexity scoring function is based on a statistical model of k-mer count distribution and our algorithm is practically close to O(wL) in time complexity, enabling the efficient identification of LC strings in long context windows.

## 2. Methods

Similar to SDUST (Morgulis et al., 2006), we define the complexity of a DNA string with a function of the k-mer counts of the string. In this section, we will first model the k-mer count distribution of random strings. We will then describe the complexity scoring function and the condition on bounding LC substrings in a long string. We will compare our method to SDUST in the end.

### 2.1. Notations

Let Σ={A,C,G,T} be the DNA alphabet, $x\in {\text{Σ}}^{*}$ is a DNA string and |x| is its length. $t\in {\text{Σ}}^{k}$ is a k-mer. For |x|≥k, $c_{x}(t)$ is the occurrence of k-mer t in x; $\ell (x)=\sum_{t}c_{x}(t)=|x|-k+1$ is the total number of k-mers in x. ${\vec{c}}_{x}$ denotes the count array over all k-mers.

In this article, we assume there is one long genome string of length L. We use closed interval [i,j] to denote the substring starting at i and ending at j, including the end points i and j. We may use “interval” and “subsequence” interchangeably.

### 2.2. Modeling k-mer counts

Suppose symbols in Σ all occur at equal frequency. Then for all k-mer t, $c_{x}(t)∼\text{Poisson}(\lambda )$ where $\lambda =\ell (x)/4^{k}$. Let

$$p(n|\lambda )≜\frac{\lambda^{n}}{n!}e^{-\lambda }$$

be the probability mass function of Poisson distribution. Notably, although $c_{x}(t)\leq \ell (x)$, given that ℓ(x)≫1 in practice,

$$p(\ell |\lambda )\approx \frac{e^{-\lambda }}{\sqrt{2\pi \ell }}\cdot {\left(\frac{e}{4^{k}}\right)}^{\ell }\ll 1$$

with the Sterling formula −p(ℓ∣λ) is very close to 0. This suggests Poisson remains a good approximation.

The composite probability of string x can be modeled by

$$P\left({\vec{c}}_{x}\right)=\prod_{t\in {\text{Σ}}^{k}}p\left(c_{x}(t)|\lambda \right)$$

We have

(1) $$\log P\left({\vec{c}}_{x}\right)=4^{k}\lambda (\log\lambda -1)-\sum_{t}\log c_{x}(t)!$$

To get an intuition about $P\left({\vec{c}}_{x}\right)$, suppose $\ell (x)\ll 4^{k}$. In this case, $c_{x}(t)$ will be mostly 0 or 1 for a random string and the last term in Eq. (1) will be close to 0. Given an LC string of the same length, we will see more $c_{x}(t)$ of 2 or higher, which will reduce $\log P\left({\vec{c}}_{x}\right)$. Thus the probability of an LC string is lower under this model.

Although $\log P\left({\vec{c}}_{x}\right)$ can be used to compare the complexity of strings of the same length, it does not work well for strings of different lengths because $\log P\left({\vec{c}}_{x}\right)$ decreases with ℓ(x). We would like to scale it to $Q\left({\vec{c}}_{x}\right)$ such that Q approaches 0 given a random string. We note that on the assumption of equal base frequency, the average of $\log P\left({\vec{c}}_{x}\right)$ can be approximated to

$$\begin{array}{l} H(\lambda )≜\sum_{\vec{c}}P(\vec{c})\cdot \sum_{t}\log p\left(c_{t}|\lambda \right)\\ \;=4^{k}\sum_{n=0}^{\infty }p(n|\lambda )\log p(n|\lambda )\\ \;=4^{k}\lambda (\log\lambda -1)-4^{k}e^{-\lambda }\sum_{n=0}^{\infty }\log n!\cdot \frac{\lambda^{n}}{n!} \end{array}$$

which is the negative entropy of P. We can thus define

$$Q\left({\vec{c}}_{x}\right)≜H(\lambda )-\log P\left({\vec{c}}_{x}\right)=\sum_{t}\log c_{x}(t)!-f\left(\frac{\ell (x)}{4^{k}}\right)$$

where

$$f(\lambda )≜4^{k}e^{-\lambda }\sum_{n=0}^{\infty }\log n!\cdot \frac{\lambda^{n}}{n!}$$

$Q\left({\vec{c}}_{x}\right)$ is higher for LC string x.

### 2.3. Scoring low-complexity intervals

To put a threshold on the complexity, we finally use the following function to score string complexity:

(2) $$S\left({\vec{c}}_{x}\right)≜Q\left({\vec{c}}_{x}\right)-T\cdot \ell (x)=\sum_{t}\log c_{x}(t)!-T\cdot \ell (x)-f\left(\frac{\ell (x)}{4^{k}}\right)$$

Threshold T controls the level of complexity in the output. It defaults to 0.6, less than log2. If $S\left({\vec{c}}_{x}\right)>0$, x is considered to contain an LC substring. Note that we often do not want to classify the entire x as an LC substring in this case because the concatenation of a highly repetitive sequence and a random sequence could still lead to a positive score.

Recall that we may use close intervals to represent substrings. For convenience, we write $S\left({\vec{c}}_{\left[i,j\right]}\right)$ as S(i,j). In implementation, we precalculate $f\left(\ell /4^{k}\right)$ and introduce

$$\begin{array}{l} U(i,j)≜\sum_{t}\log c_{\left[i,j\right]}(t)!-T\cdot \ell ([i,j])\\ \;=U(i,j-1)+\log c_{\left[i,j\right]}([j-k+1,j])-T \end{array}$$

We can thus compute the complexity scores of all prefixes of [i,j] by scanning each base in the interval from left to right; we can similarly compute all suffix scores from right to left.

### 2.4. Finding low-complexity regions

We say x is a *perfect LC string* (or *perfect LC interval*) if $S\left({\vec{c}}_{x}\right)>0$ and no substring of x is scored higher than $S\left({\vec{c}}_{x}\right)$; say x is a *good LC string* (or *good LC interval*) if $S\left({\vec{c}}_{x}\right)>0$ and no prefix or suffix of x is scored higher than $S\left({\vec{c}}_{x}\right)$. We can use U(i,j) above to test if [i,j] is a good LC interval in linear time. If we apply this method to all intervals up to w in length (5000bp by default), we can find LC regions of context length up to w in $O\left(w^{2}L\right)$ time. The union of all good LC intervals marks the LC regions in a genome.

**Algorithm 1:** Find LC interval ending at j

1:	**procedure** FindStart$\left(k,w,T,j,c^{\prime }\right)$
2:	B← Backward$\left(k,w,T,j,c^{\prime }\right)$
3:	$j_{\max}^{\prime }\leftarrow -1$
4:	**for** $\left(i,v^{\prime }\right)\in B$ in the ascending order of i **do**
5:	**continue if** $i<j_{\max}^{\prime }$ ▷ this is an approximation
6:	$j^{\prime }\leftarrow$ Forward $\left(k,T,i,j,v^{\prime }\right)$
7:	**return** i **if** $j^{\prime }=j$ ▷ [i,j] is a good LC interval
8:	$j_{\max}^{\prime }\leftarrow \max\left(j_{\max}^{\prime },j^{\prime }\right)$
9:	**end for**
10:	**return** − 1 ▷ No good LC interval ending at j
11:	**end procedure**
12:	**procedure** Backward$\left(k,w,T,j,c^{\prime }\right)$
13:	u←0; $v_{0}\leftarrow -1$; $u^{\prime }\leftarrow 0$
14:	$v_{\max}\leftarrow 0$; $i_{\max}\leftarrow -1$; c←[0]
15:	B←∅
16:	**for** i←j **to** max(j−w+1,k−1) **do** ▷ i is descending
17:	t←[i−k+1,i] ▷ the k-mer ending at i
18:	c[t]←c[t]+1
19:	u←u+log(c[t])−T
20:	$v\leftarrow u-f\left((j-i+1)/4^{k}\right)$ ▷ v=S(i−k+1,j)
21:	**if** $v<v_{0}$ **and** $v_{0}=v_{\max}$ **then**
22:	$B\leftarrow B\cup \left\{\left(i+1,v_{\max}\right)\right\}$ ▷ a candidate start pos
23:	**else if** $v\geq v_{\max}$ **then**
24:	$v_{\max}\leftarrow v$; $i_{\max}\leftarrow i$
25:	**else if** $i_{\max}<0$ **then**
26:	$u^{\prime }\leftarrow u^{\prime }+\log\left(c^{\prime }[t]\right)-T$ ▷ $c^{\prime }[t]≜c_{\left[j-w+1,j\right]}(t)$
27:	**break if** $u^{\prime }<0$ ▷ Forward() wouldn’t reach j
28:	**end if**
29:	$v_{0}\leftarrow v$
30:	**end for**
31:	$B\leftarrow B\cup \left\{\left(i_{\max},v_{\max}\right)\right\}$ **if** $i_{\max}\geq 0$
32:	**return** B
33:	**end procedure**
34:	**procedure** Forward$\left(k,T,i_{0},j,v_{\max}^{\prime }\right)$
35:	u←0; $v_{\max}\leftarrow 0$; $i_{\max}\leftarrow -1$; c←[0]
36:	**for** $i\leftarrow i_{0}$ **to** j **do**
37:	t←[i−k+1,i]
38:	c[t]←c[t]+1
39:	u←u+log(c[t])−T
40:	$v\leftarrow u-f\left(\left(i-i_{0}+1\right)/4^{k}\right)$
41:	**if** $v\geq v_{\max}$ **then**
42:	$v_{\max}\leftarrow v$; $i_{\max}\leftarrow i$
43:	**end if**
44:	**break if** $v>v_{\max}^{\prime }$
45:	**end for**
46:	**return** $i_{\max}$
47:	**end procedure**

Algorithm 1 shows a faster way to find a good LC interval ending at j. Function Backward() scans backwardly from j to j−w+1 to collect candidate start positions (line 22). Variable v is the complexity score of suffix [i−k+1,j] (line 20). By the definition of good LC interval, i can only be a candidate start if v is no less than all the suffixes visited before (line 21). We also ignore a candidate start i if S(i,j)<S(i−1,j) because if [i−1,j] is not a good LC interval, there must exist $i^{\prime }>i$ such that $S\left(i^{\prime },j\right)>S(i-1,j)<S(i,j)$, so [i,j] would not be a good LC interval, either. In addition, if suffix [i,j] is enriched with k-mers unique in the full window [j−w+1,j], [i,j] will not be a good LC interval (line 27) as there will exist $j^{\prime }<j$ such that $S\left(i,j^{\prime }\right)>S(i,j)$. The time complexity of Backward() is O(w).

Given a candidate start position i, function Forward() returns $j^{\prime }=\text{argma}{\text{x}}_{i<j^{\prime }\leq j}S\left(i,j^{\prime }\right)$. [i,j] will be a good LC interval if and only if $j^{\prime }=j$ (Line 7). We call Forward() in the ascending order of candidate start positions (line 4). We may skip a start position if it is contained in an interval found from previous Forward() calls (line 5). This is an approximation as it is possible for a good LC interval to start in another good interval. An alternative heuristic is to only apply Forward() to the smallest candidate start in B. This leads to a guaranteed O(w) with FindStart(). In practice, the two algorithms have almost identical runtime. We use Algorithm 1 in longdust as it is closer to the exact algorithm.

Function FindStart() finds the longest good LC interval ending at one position. We apply the function to every position in the genome to find all good LC intervals. We can skip j if [j−k+1,j] is unique in [j−w+1,j] because the forward pass would not reach j in this case. We also introduce a heuristic to extend a good LC interval [j−w,j−1] to [j−w+1,j] without calling FindStart(). We additionally use an X-drop heuristic (Altschul et al., 1997) to avoid connecting two good LC intervals occasionally.

The overall longdust algorithm is inexact and may result in slightly different LC regions (21kb out of 278Mb in T2T-CHM13). We run the algorithm on both the forward and the reverse strand of the input sequences and merge the resulting intervals. The default longdust output is strand symmetric.

### 2.5. Comparison to SDUST

SDUST (Morgulis et al., 2006) uses the following complexity scoring function:

$$S_{S}\left({\vec{c}}_{x}\right)=\frac{1}{\ell (x)}\sum_{t}\frac{c_{x}(t)\left(c_{x}(t)-1\right)}{2}-T$$

This function grows linearly with ℓ(x) for $\ell (x)\geq 4^{k}$, while our scoring function grows more slowly in the logarithm scale. The SDUST function is more likely to classify longer sequences as LC.

Furthermore, SDUST looks for perfect LC intervals rather than good LC intervals like longdust. It cannot test whether an interval is perfect in linear time. Instead, SDUST maintains the complete list of perfect intervals in window [j−w+1,j] and tests a new candidate interval against the list. The FindStart() equivalent of SDUST is $O\left(w^{3}\right)$ in time, impractical for long windows. SDUST hardcodes k=3 and uses w=64 by default for acceptable performance.

### 2.6. Scoring with Shannon entropy

Let $p_{x}(t)=c_{x}(t)/\ell (x)$. The Shannon entropy of string x is

$$H\left({\vec{c}}_{x}\right)≜-\sum_{t}p_{x}(t)\log p_{x}(t)=\log\ell (x)-\frac{1}{\ell (x)}\sum_{t}c_{x}(t)\log c_{x}(t)$$

When $\ell (x)\leq 4^{k}$, $H\left({\vec{c}}_{x}\right)$ reaches the maximum value of logℓ(x) at $p_{x}(t)=1/\ell (x)$. $H\left({\vec{c}}_{x}\right)$ also grows with ℓ(x). For $\ell (x)\leq 4^{k}$, define

$$S_{E}\left({\vec{c}}_{x}\right)≜\log\ell (x)-H\left({\vec{c}}_{x}\right)-T=\frac{1}{\ell (x)}\sum_{t}c_{x}(t)\log c_{x}(t)-T$$

We adapted longdust for $S_{E}$ and found using $S_{E}$ is more than twice as slow. We suspected some longdust heuristics did not work well with $S_{E}$, but we did not investigate further.

## 3. Results

### 3.1. Low-complexity regions in T2T-CHM13

We applied longdust, SDUST v0.1 (Morgulis et al., 2006), pytrf v1.4.2 (Du et al., 2025), TRF v4.10 (Benson, 1999), TANTAN v51 (Frith, 2011), and ULTRA v1.20 (Olson and Wheeler, 2024) to the T2T-CHM13 human genome (Nurk et al., 2022). Command lines for TRF, TANTAN and ULTRA were adopted from Olson and Wheeler (2024), with the maximum period set to 500 (Table 1). Notably, TRF would not finish in days with the default option −l. An even larger −l helps performance at the cost of memory.

**Fig. 1.**
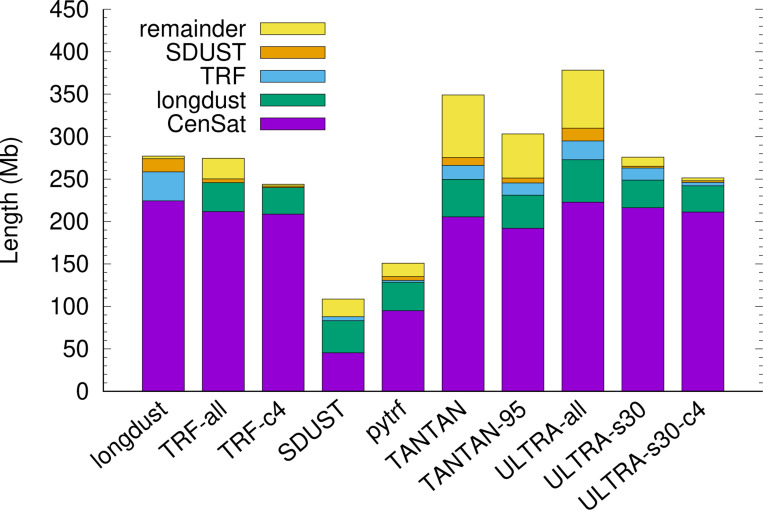
Lengths of low-complexity regions. Low-complexity (LC) regions identified by each tool are first intersected with centromeric satellite annotation. The remainder is then intersected with longdust, TRF and SDUST in order. There are no overlaps between stacks. The total height is the length of LC regions found by each tool. Alternative settings – “TRF-c4”: requiring ≥ 4 copies of the repeat unit; “ULTRA-s30”: requiring score ≥ 30 in ULTRA output; “TANTAN-95”: run with “-s.95” for more stringent output.

Longdust finds 277.1Mb of LC regions with 224.3Mb overlapping with centromeric satellite annotated by the telomere-to-telomere (T2T) consortium (Fig. 1). Of the remaining 52.7Mb, 34.1Mb overlaps with TRF; 15.4Mb of the remainder (18.6Mb) is found by SDUST. Only 3.2Mb is left, suggesting most longdust LC regions fall in centromeres or are found by TRF or SDUST.

TRF, the most popular tandem repeat finder, finds 274.5Mb of tandem repeats, 244.0Mb of which have ≥ 4 copies of repeat units. 97.9% of the 244.0Mb are identified by longdust. TRF additionally reports tandem repeats with < 4 repeat units. Only 14.8% of them overlap with longdust results. Longdust misses repeats with low copy numbers. In fact, under the default threshold T=0.6, the minimum numbers of exact copies longdust can find is approximately:

$$3+\frac{k-1}{r}+\frac{3T-\log2-\log3}{\log4-T}\approx 3.01+\frac{k-1}{r}$$

This assumes the repeat unit length r>k, f(⋅)=0 and all k-mers are unique within the repeat unit.

As to other tools, ULTRA outputs the largest tandem repeat regions (Fig. 1). Nevertheless, if we raise its score threshold to 30, the total region length is similar to that of TRF. TANTAN also reports many regions not found by longdust, TRF or SDUST. Increasing its probability threshold only marginally change the relative portions. Pytrf cannot effectively identify alpha satellite with ∼170bp repeat units, even though it was set to find tandem repeats with unit up to 500bp. SDUST is the only other method that looks for LC regions not limited to tandem repeats. With a 64bp window size, it naturally misses tandem repeats with long units, including all alpha satellite.

### 3.2. Low-complexity regions in a gorilla genome

The near T2T gorilla genome (AC:GCF_029281585.2; Yoo et al., 2025) is 3546Mb in size, 428Mb larger than the human T2T-CHM13 genome. We ran longdust on the gorilla genome for 1.4 hours and found 656.8Mb of LC regions. That is 379.7Mb larger than the LC regions in T2T-CHM13. The genome size difference is primarily driven by LC regions.

To further confirm this observation, we extracted 298.8Mb of regions in the gorilla genome that are ≥ 10kb in length without any 51-mer exact matches to 472 human genomes (Li, 2024). 99.7% of them are marked as LC regions by longdust and none of them are alpha satellite. 95.8% of these gorilla-specific regions are distributed within 15Mb from telomeres, broadly in line with Yoo et al. (2025). We also ran TRF on the gorilla genome. It did not finish in 30 hours even with option “-l20” which is supposed to reduce runtime.

## 4. Discussions

Implemented in the C programming language, longdust is a fast and lightweight command-line tool for identifying low-complexity regions. It rarely finds LC regions not reported by TRF plus SDUST and can recover more tandem repeats with ≥ 4 copies of repeat units. Longdust provides basic APIs in C and can also be used as a programming library.

From the theoretical point of view, longdust uses an approximate algorithm. It tests LC intervals ending at each position with Algorithm 1, but has not sufficiently exploited dependencies between positions. It will be interesting to see whether there is an exact O(wL) algorithm under the longdust formulation or a meaningful alternative formulation that leads fast implementations.

A major practical limitation of longdust is the restricted window size. The genome of Woodhouse’s scrub jays, for example, contains satellite with a 12kb repeat unit (Edwards et al., 2025). This would be missed by longdust under the default setting. Increasing the window size would make longdust considerably slower. This is partly due to the O(wL) time complexity and partly due to the speedup strategies in longdust that are more effective given $\ell (x)\ll 4^{k}$. It would be ideal to have an algorithm that remains efficient given large windows or, better, does not require specified window sizes.

## Figures and Tables

**Table 1.** Command lines and resource usage for T2T-CHM13

Tool	CPU time	Mem (G)	Command line
longdust	1h3m	0.47	(default)
SDUST	4m15s	0.23	-t30
pytrf	2h39m	0.70	-M500
TRF	12h52m	7.49	2 7 7 80 10 50 500 -l12
TANTAN	32m50s	1.28	-w500 -s.85
ULTRA	146h4m	33.31	-p500 -t16

Performance measured on a Linux server equipped with Intel Xeon Gold 6130 CPU and 512GB memory.

## Data Availability

https://github.com/lh3/longdust
